# A Bayesian View on Cryo-EM Structure Determination

**DOI:** 10.1016/j.jmb.2011.11.010

**Published:** 2012-01-13

**Authors:** Sjors H.W. Scheres

**Affiliations:** MRC Laboratory of Molecular Biology, Hills Road, Cambridge CB2 0QH, UK

**Keywords:** 3D, three-dimensional, cryo-EM, cryo-electron microscopy, SNR, signal-to-noise ratio, ML, maximum likelihood, MAP, *maximum a posteriori*, 2D, two-dimensional, FSC, Fourier shell correlation, EF-G, elongation factor G, CTF, contrast transfer function, cryo-electron microscopy, three-dimensional reconstruction, *maximum a posteriori* estimation

## Abstract

Three-dimensional (3D) structure determination by single-particle analysis of cryo-electron microscopy (cryo-EM) images requires many parameters to be determined from extremely noisy data. This makes the method prone to overfitting, that is, when structures describe noise rather than signal, in particular near their resolution limit where noise levels are highest. Cryo-EM structures are typically filtered using *ad hoc* procedures to prevent overfitting, but the tuning of arbitrary parameters may lead to subjectivity in the results. I describe a Bayesian interpretation of cryo-EM structure determination, where smoothness in the reconstructed density is imposed through a Gaussian prior in the Fourier domain. The statistical framework dictates how data and prior knowledge should be combined, so that the optimal 3D linear filter is obtained without the need for arbitrariness and objective resolution estimates may be obtained. Application to experimental data indicates that the statistical approach yields more reliable structures than existing methods and is capable of detecting smaller classes in data sets that contain multiple different structures.

## Graphical Abstract

**Figure:**
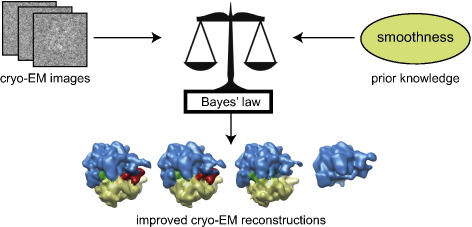

**Highlights:**

► Limiting overfitting in cryo-EM reconstruction requires user expertise. ► A Bayesian approach may limit overfitting without arbitrary decisions, providing novel insights in the Wiener filter for 3D reconstruction, yielding better structures and improved classifications, and contributing to increase the objectivity in cryo-EM reconstruction.

## Introduction

With recent reports on near-atomic-resolution (i.e., 3–4 Å) structures for several icosahedral viruses and resolutions in the range of 4–6 Å for complexes with less or no symmetry, cryo-electron microscopy (cryo-EM) single-particle analysis has entered the exciting stage where it may be used for *de novo* generation of atomic models.^1^ However, the observation that reported resolutions vary significantly for maps with otherwise similar features^2^ is an indication that existing reconstruction methods suffer from different degrees of overfitting. Overfitting occurs when the reconstruction describes noise instead of the underlying signal in the data, and often, these noisy features are enhanced during iterative refinement procedures. Thereby, overfitting is not merely an issue of comparing the resolution of one reconstruction with another but represents a major obstacle in the objective analysis of cryo-EM maps. In particular, without a useful cross-validation tool, such as the free *R*-factor in X-ray crystallography,^3^ overfitting may remain undetected and a map may be interpreted at a resolution where the features are mainly due to noise.

At the heart of the problem lies the indirectness of the experimental observations. A reasonably good model is available for the image formation process. Given a three-dimensional (3D) structure, this so-called forward model describes the appearance of the experimental images. However, the problem of single-particle reconstruction is the inverse one and is much more difficult to solve. The structure determination task is further complicated by the lack of information about the relative orientations of all particles and, in the case of structural variability in the sample, also their assignment to a structurally unique class. These data are lost during the experiment, where molecules in distinct conformations coexist in solution and adopt random orientations in the ice. In mathematics, this type of problem where part of the data is missing is called *incomplete*. Moreover, because the electron exposure of the sample needs to be strictly limited to prevent radiation damage, experimental cryo-EM images are extremely noisy. The high levels of noise together with the incompleteness of the data mean that cryo-EM structures are not fully determined by the experimental data and therefore prone to overfitting. In mathematical terms, the cryo-EM structure determination problem is *ill-posed*.

Ill-posed problems can be tackled by regularization, where the experimental data are complemented with external or prior information so that the two sources of information together fully determine a unique solution. A particularly powerful source of prior information about cryo-EM reconstructions is smoothness. Because macromolecules consist of atoms that are connected through chemical bonds, the scattering potential will vary smoothly in space, especially at less than atomic resolution. The concept of imposing smoothness to prevent overfitting is widely used in the field through a variety of *ad hoc* filtering procedures. By limiting the power of the reconstruction at those frequencies where the signal-to-noise ratio (SNR) is low, these filters impose smoothness on the reconstructed density in real space. Traditionally, filtering procedures have relied on heuristics, that is, to some extent, existing implementations are all based on arbitrary decisions. Although potentially highly effective (and this is illustrated by the high-resolution structures mentioned above), the heuristics in these methods often involve the tuning of free parameters, such as low-pass filter shape and effective resolution (e.g., see Ref. 4). Thereby, the user (or, in some cases, the programmer) becomes responsible for the delicate balance between getting the most out of the data and limiting overfitting, which ultimately may lead to subjectivity in the structure determination process.

Recent attention for statistical image processing methods^5^ could be explained by a general interest in reducing the amount of heuristics in cryo-EM reconstruction procedures. Rather than combining separate steps of particle alignment, class averaging, filtering, and 3D reconstruction, each of which may involve arbitrary decisions, the statistical approach seeks to maximize a single probability function. Most of the statistical methods presented thus far have optimized a likelihood function, that is, one aims to find the model that has the highest probability of being the correct one in the light of the observed data. This has important theoretical advantages, as the maximum likelihood (ML) estimate is asymptotically unbiased and efficient. That is, in the limit of very large data sets, the ML estimate is as good as or better than any other estimate of the true model (see Ref. 6 for a recent review on ML methods in cryo-EM). In practice, however, data sets are not very large, and also in the statistical approach, the experimental data may need to be supplemented with prior information in order to define a unique solution. In Bayesian statistics, regularization is interpreted as imposing prior distributions on model parameters, and the ML optimization target may be augmented with such prior distributions. Optimization of the resulting posterior distribution is called *regularized likelihood* optimization, or *maximum a posteriori* (MAP) estimation (see Ref. 7).

In this paper, I will show that MAP estimation provides a self-contained statistical framework in which the regularized single-particle reconstruction problem can be solved with only a minimal amount of heuristics. As a prior, I will use a Gaussian distribution on the Fourier components of the signal. Neither the use of this prior nor that of the Bayesian treatment of cryo-EM data is a new idea. Standard textbooks on statistical inference use the same prior in a Bayesian interpretation of the commonly used Wiener filter (e.g., see Ref. 7, pp. 549–551), and an early mention of MAP estimation with a Gaussian prior in the context of 3D EM image restoration was given by Carazo.^8^ Nevertheless, even though these ideas have been around for many years, the Bayesian approach has thus far not found wide-spread use in 3D EM structure determination (see Ref. 9 for a recent application). This limited use contrasts with other methods in structural biology. Recently, Bayesian inference was shown to be highly effective in NMR structure determination,^10^ while the Bayesian approach was introduced to the field of X-ray crystallography many years ago^11^ and MAP estimation is now routinely used in crystallographic refinement.^12^

In what follows, I will first describe some of the underlying theory of existing cryo-EM structure determination procedures to provide a context for the statistical approach. Then, I will derive an iterative MAP estimation algorithm that employs a Gaussian prior on the model in Fourier space. Because statistical assumptions about the signal and the noise are made explicit in the target function, straightforward calculus in the optimization of this target leads to valuable new insights into the optimal linear (or Wiener) filter in the context of 3D reconstruction and the definition of the 3D SNR in the Fourier transform of the reconstruction. Moreover, because the MAP algorithm requires only a minimum amount of heuristics, arbitrary decisions by the user or the programmer may be largely avoided, and objectivity may be preserved. I will demonstrate the effectiveness of the statistical approach by application to three cryo-EM data sets and compare the results with those obtained using conventional methods. Apart from overall improvements in the reconstructed maps and the ability to detect smaller classes in structurally heterogeneous data sets, the statistical approach reduces overfitting and provides reconstructions with more reliable resolution estimates.

## Theory

### Conventional methods

Many different procedures have been implemented to determine 3D structures from cryo-EM projection data. The following does not seek to describe all of them but, rather, aims to provide an accessible introduction to the Bayesian approach described below. For an extensive review of existing cryo-EM methods, the reader is referred to the book by Frank^4^ or to the more recent volumes 481–483 of the book series *Methods in Enzymology*.^13^

Almost all existing implementations for cryo-EM structure determination employ the so-called weak-phase object approximation, which leads to a linear image formation model in Fourier space:

(1) $$X_{ij}={\text{CTF}}_{ij}\sum_{l=1}^{L}P_{jl}^{\phi }V_{l}+N_{ij}$$

where:

• *X*_*ij*_ is the *j*th component, with *j* = 1,…,*J*, of the two-dimensional (2D) Fourier transform of the *i*th experimental image X_*i*_, with *i* = 1,…,*N*.
• CTF_*ij*_ is the *j*th component of the contrast transfer function for the *i*th image. Some implementations, such as EMAN,^14^ include an envelope function on the contrast transfer function (CTF) that describes the fall-off of signal with resolution. Other implementations, such as FREALIGN,^15^ ignore envelope functions at this stage and correct for signal fall-off through *B*-factor sharpening of the map after refinement.^16^ The latter intrinsically assumes identical CTF envelopes for all images.
• *V*_*l*_ is the *l*th component, with *l* = 1,…,*L*, of the 3D Fourier transform *V* of the underlying structure in the data set. Estimating *V* is the objective of the structure determination process. For the sake of simplicity, only the structurally homogeneous case is described here. Nevertheless, Eq. (1) may be expanded to describe structural heterogeneity, that is, data sets that contain more than one underlying 3D structure, by adding a subscript: *V*_*k*_, with *k* = 1,…,*K*. Often, *K* is assumed to be known,^17^ so that each experimental image can be described as a projection of one of *K* different structures, each of which needs to be estimated from the data.
• **P**^ϕ^ is a *J*  × *L* matrix of elements **P**_*jl*_^ϕ^. The operation $\sum_{l=1}^{L}P_{jl}^{\phi }V_{l}$ for all *j* extracts a slice out of the 3D Fourier transform of the underlying structure, and Φ defines the orientation of the 2D Fourier transform with respect to the 3D structure, comprising a 3D rotation and a phase shift accounting for a 2D origin offset in the experimental image. Similarly, the operation $\sum_{j=1}^{J}P^{\phi_{lj}^{T}}X_{ij}$ for all *l* places the 2D Fourier transform of an experimental image back into the 3D transform. According to the projection-slice theorem, these operations are equivalent to the real-space projection and “back-projection” operations. Some implementations calculate (back)-projections in real space, such as XMIPP;^18^ other implementations, such as FREALIGN,^15^ perform these calculations in Fourier space.
• *N*_*ij*_ is noise in the complex plane. Although explicit assumptions about the statistical characteristics of the noise are not often reported, commonly employed Wiener filters and cross-correlation goodness-of-fit measures rely on the assumption that the noise is independent and Gaussian distributed.

After selection of the individual particles from the digitized micrographs, the experimental observations comprise *N* images *X*_*i*_. From the micrographs, one may also calculate the CTFs, which are then kept constant in most procedures. The estimation of *V* from all *X*_*i*_ and CTF_*i*_ is then typically accomplished by an iterative procedure (called refinement) that requires an initial, often low-resolution, 3D reference structure *V*^(0)^. As this paper is primarily concerned with refinement, the reader is referred to the books mentioned above for more information about how these starting models may be obtained. At every iteration (*n*) of the refinement process, projections of *V*^(*n*)^ are calculated for many different orientations ϕ and compared with each of the experimental images. Based on some goodness-of-fit measure, an optimal orientation ϕ_*i*_^⁎^ is assigned to each image. All images are then combined into a 3D reconstruction that yields the updated model *V*^(*n* + 1)^. Many different reconstruction algorithms are available, but their description falls outside the scope of this paper (again, the reader is referred to the books mentioned above). In what follows, I will focus on a class of algorithms that has been termed direct Fourier inversion and will mostly ignore complications due to interpolations and nonuniform sampling of Fourier space. The update formula for *V* may then be given by (for all *l*):

(2) $$V_{l}^{\left(n\text{+1}\right)}=\frac{\sum_{i=1}^{N}{\sum_{j=1}^{J}P^{\phi_{i}^{*}}}_{lj}^{T}{\text{CTF}}_{ij}X_{ij}}{\sum_{i=1}^{N}{\sum_{j=1}^{J}P^{\phi_{\text{i}}^{*}}}_{lj}^{T}{\text{CTF}}_{ij}^{\text{2}}}$$

and this procedure is typically repeated until changes in *V* and/or ϕ*_i_^⁎^* become small. It is important to realize that this refinement is a local optimization procedure that is prone to becoming stuck in local minima (and the same is true for the statistical approach outlined below). Consequently, the initial reference structure *V*^(0)^ may have an important effect on the outcome of the refinement, as wrong initial models could lead to incorrect solutions. Still, if one ignores local minima and if the goodness-of-fit measure used in the assignment of all ϕ*_i_^⁎^* is a least-squares or cross-correlation criterion, then one could argue that this procedure provides a least-squares estimate of the true 3D structure.

However, as explained in Introduction, the observed data alone are not sufficient to uniquely determine the correct solution. Consequently, without the inclusion of additional, prior information *V* may become very noisy, especially at frequencies where many CTFs have zero or small values and at high frequencies where SNRs are lowest. Many existing implementations reduce the noise levels in *V* by means of a so-called Wiener filter. This image restoration method is based on minimization of the mean-square error between the estimate and the true signal and effectively regularizes the ill-posed problem by introducing prior knowledge about the correlation structure of the signal and the noise.^8^ Most often, Wiener filter expressions are given for the case of 2D averaging, as relatively little work is published on the Wiener filter for 3D reconstruction.^19^ If one assumes that both the signal and the noise are independent and Gaussian distributed with power spectra τ^2^(υ) for the signal and power spectra σ_*i*_^2^(υ) for the noise, with *v* being the frequency, then (variants of) the following expression for the Wiener filter for 2D averaging are often reported:^20^

(3) $$A_{j}=\frac{\sum_{i=1}^{N}\frac{\tau^{2}\left(\upsilon \right)}{\sigma^{2}\left(\upsilon \right)}{\text{CTF}}_{ij}X_{ij}}{\sum_{i=1}^{N}\frac{\tau^{2}\left(\upsilon \right)}{\sigma^{2}\left(\upsilon \right)}{\text{CTF}}_{ij}^{\text{2}}+1}$$

where *A*_*j*_ is the *j*th component of the 2D Fourier transform of average image *A*.

The addition of one in the denominator of Eq. (3) reduces noise by reducing the power in the average for those Fourier components where $\sum_{i=1}^{N}\frac{\tau^{2}(\upsilon )}{\sigma^{2}(\upsilon )}{\text{CTF}}_{ij}^{\text{2}}$ is small. One could discern two effects of the Wiener filter, the first of which is recognized much more often than the second. (i) The Wiener filter corrects for the CTF, that is, A will represent the original signal, unaffected by the CTF. (ii) The Wiener filter also acts as a low-pass filter. If one ignores the CTF in the Wiener filter expression by setting all CTF_*ij*_ in Eq. (3) equal to 1, then a filter remains that solely depends on the resolution-dependent SNR $\left(\frac{\tau^{2}(\upsilon )}{\sigma^{2}(\upsilon )}\right)$. Since the SNR in cryo-EM images of macromolecular images typically drops quickly with resolution (e.g., see Fig. 3a), this will effectively be a low-pass filter.

In the case of 3D reconstruction, consensus about the Wiener filter has not yet been reached, and existing implementations have worked around this problem by employing a variety of *ad hoc* procedures.^19^ Two common approximations are to apply Wiener filtering to 2D (class) averages and/or to assume that $\frac{\tau^{2}(\upsilon )}{\sigma^{2}(\upsilon )}$ is a constant, the so-called Wiener constant. Examples of these two approximations may be found in EMAN^14^ and FREALIGN,^15^ respectively. If one assumes that the SNR is a constant 1/*C*, then 3D reconstruction with Wiener filtering has been expressed as (e.g., see Ref. 15):

(4) $$V_{l}^{\left(n\text{+1}\right)}=\frac{\sum_{i=1}^{N}{\sum_{j=1}^{J}P^{\phi_{i}^{*}}}_{lj}^{T}{\text{CTF}}_{ij}X_{ij}}{\sum_{i=1}^{N}{\sum_{j=1}^{J}P^{\phi_{\text{i}}^{*}}}_{lj}^{T}{\text{CTF}}_{ij}^{\text{2}}+C}$$

In many software packages, the heuristics in the Wiener filter implementation have resulted in additional free parameters, such as the Wiener constant (*C*). Moreover, as existing implementations typically fail to adequately reproduce the low-pass filtering effect of the true Wiener filter, it is common practice to apply *ad hoc* low-pass filters to *V* during the iterative refinement. This typically involves the tuning of even more parameters, such as effective resolution and filter shape. Suboptimal use of these arbitrary parameters may lead to the accumulation of noise in the reconstructed density and overfitting of the data. Consequently, a certain level of expertise is typically required to obtain the optimal estimate of *V*, which may ultimately lead to subjectivity in the cryo-EM structure determination process.

### A Bayesian view

The statistical approach explicitly optimizes a single target function. Imagining an ensemble of possible solutions, the reconstruction problem is formulated as finding the model with parameter set Θ that has the highest probability of being the correct one in the light of both the observed data X and the prior information Y. According to Bayes' law, this so-called posterior distribution factorizes into two components:

(5) P(Θ|X,Y)∝P(X|Θ,Y)P(Θ|Y)

where the *likelihood P*(X|Θ,Y) quantifies the probability of observing the data given the model, and the *prior P*(Θ|Y) expresses how likely that model is given the prior information. The model Θ̂ that optimizes *P*(Θ|X,Y) is called the MAP estimate. [Note that previously discussed ML methods optimize *P*(X|Θ,Y).]

The statistical approach employs the same image formation model as described in Eq. (1) but explicitly assumes that all noise components *N*_*ij*_ are independent and Gaussian distributed. The variance σ*_ij_*^2^ of these noise components is unknown and will be estimated from the data. Variation of σ*_ij_*^2^ with resolution allows the description of nonwhite or colored noise. The assumption of independence in the noise allows the probability of observing an image given its orientation and the model to be calculated as a multiplication of Gaussians over all its Fourier components,^21^ so that:

(6) $$P\left(X_{i}|\phi ,\Theta ,Y\right)=\prod_{j=1}^{J}\frac{1}{2\pi \sigma_{ij}^{2}}\text{exp}\left(\frac{{\left|X_{ij}-{\text{CTF}}_{ij}\sum_{l=1}^{L}P_{jl}^{\phi }V_{l}\right|}^{2}}{-2\sigma_{ij}^{2}}\right)$$

The correct orientations ϕ for all images are not known. They are treated as hidden variables and are integrated out. The corresponding marginal likelihood function of observing the entire data set X is then given by:

(7) $$P\left(\text{X}|\Theta ,\text{Y}\right)=\prod_{i=1}^{N}\int_{\phi }P\left({\text{X}}_{i}|\phi ,\Theta ,\text{Y}\right)P\left(\phi |\Theta ,\text{Y}\right)\text{d}\phi$$

where *P*(ϕ|Θ,Y) expresses prior information about the distribution of the orientations. These distributions may include Gaussian distributions on the origin offsets (e.g., see Ref. 6) but their exact expression and the corresponding parameters will be ignored in what follows.

Calculation of the prior relies on the assumption of smoothness in the reconstruction. Smoothness is encoded in the assumption that all Fourier components *V*_*l*_ are independent and Gaussian distributed with zero mean and unknown variance τ*_l_*^2^, so that:

(8) $$P\left(\Theta |Y\right)=\prod_{l=1}^{L}\frac{1}{2\pi \tau_{l}^{2}}\text{exp}\left(\frac{{\left|V_{l}\right|}^{2}}{-2\tau_{l}^{2}}\right)$$

The assumption of zero-mean Fourier components of the underlying 3D structures may seem surprising at first. However, given that Fourier components may point in any (positive or negative) direction in the complex plane, their expected value in the absence of experimental data will indeed be zero. The regularizing behavior of this prior is actually through its scale parameter τ*_l_*^2^. By imposing small values of τ*_l_*^2^ on high-frequency components of *V*, one effectively limits the power of the signal at those frequencies, which acts like a low-pass filter in removing high-frequency noise, and thus imposes smoothness. Note that the explicit assumptions of independent, zero-mean Gaussian distributions for both the signal and the noise in the statistical approach are the same ones that underlie the derivation of the Wiener filter described above.

**Fig. 1:**
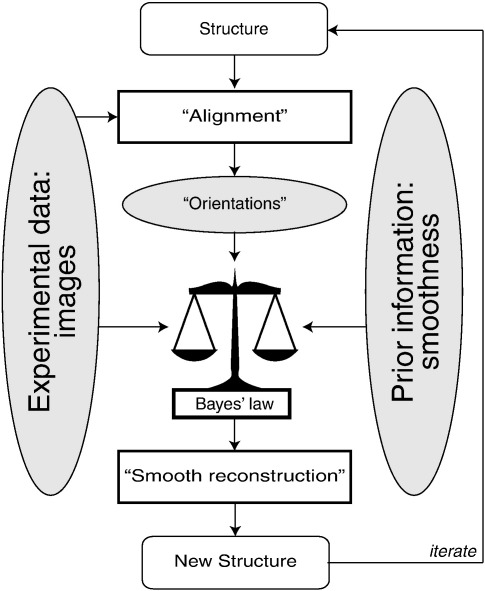
A schematic interpretation of the approach. A structure is iteratively refined through a two-step procedure. The first step, which is called *Expectation* in mathematical terms, has been labeled “Alignment.” In this step, computer-generated projections of the structure are compared with the experimental images, resulting in information about the relative orientations of the images. Orientations are not assigned in a discrete manner, but probability distributions over all possible assignments [Γ_*i*__ϕ_^(*n*^^)^] are calculated, and the sharpness of these distributions is determined by the power of the noise in the data. The second step is called *Maximization* and has been labeled “Smooth reconstruction.” In this step, the experimental images are combined with the prior information into a smooth, 3D reconstruction through Eq. (9), and updated estimates for the power of the noise and the signal in the data are obtained through Eqs. (10) and (11). The relative contributions of the data and the prior to the reconstruction are dictated by Bayes' law and depend on the power of the noise and the power of the signal in the data [see Eq. (9)]. The new structure and the updated estimates for the power of the noise and the signal are then used for a subsequent iteration. Iterations are typically stopped after a user-defined number or when the structures do not change anymore.

Eqs. (6–8) together define the posterior distribution as given in Eq. (5). For a given set of images *X*_*i*_ and their CTFs, one aims to find the best values for all *V*_*l*_, τ*_l_*^2^, and σ*_ij_*^2^. Optimization by expectation maximization^22^ yields the following algorithm (also see Fig. 1):

(9) $$V_{l}^{\left(n\text{+1}\right)}=\frac{\sum_{i=1}^{N}\int_{\phi }\Gamma_{i\phi }^{\left(n\right)}{\sum_{j=1}^{J}P}^{\phi_{lj}^{T}}\frac{{\text{CTF}}_{ij}X_{ij}}{\sigma_{ij}^{2\left(n\right)}}\text{d}\phi }{\sum_{i=1}^{N}\int_{\phi }\Gamma_{i\phi }^{\left(n\right)}{\sum_{j=1}^{J}P}^{\phi_{lj}^{T}}\frac{{\text{CTF}}_{ij}^{2}}{\sigma_{ij}^{2\left(n\right)}}\text{d}\phi +\frac{1}{\tau_{l}^{2\left(n\right)}}}$$

(10) $$\sigma_{ij}^{2\left(n\text{+1}\right)}=\frac{1}{2}\int_{\phi }\Gamma_{i\phi }^{\left(n\right)}{\left|X_{ij}-{\text{CTF}}_{ij}\sum_{l=1}^{L}P_{jl}^{\phi }V_{l}^{\left(n\right)}\right|}^{\text{2}}\text{d}\phi$$

(11) $$\tau_{l}^{2\left(n+1\right)}=\frac{1}{2}{\left|V_{l}^{\left(n+1\right)}\right|}^{2}$$

where Γ_*i*__ϕ_^(*n*)^ is the posterior probability of ϕ for the *i*th image, given the model at iteration number (*n*), which is calculated as:

(12) $$\Gamma_{i\phi }^{\left(n\right)}=\frac{P\left(X_{i}|\phi ,\Theta^{\left(n\right)},\text{Y}\right)P\left(\phi |\Theta^{\left(n\right)},\text{Y}\right)}{\int_{\phi \prime }P\left(X_{i}|\phi \prime ,\Theta^{\left(n\right)},\text{Y}\right)P\left(\phi \prime |\Theta^{\left(n\right)},\text{Y}\right)\text{d}\phi \prime }$$

Just like in related ML methods,^6^ rather than assigning an optimal orientation ϕ_*i*_^⁎^ to each image, probability-weighted integrals over all possible orientations are calculated. Apart from that, Eq. (9) bears obvious resemblance to previously reported expressions of the Wiener filter for 3D reconstruction [see Eq. (4)]. This may not come as a surprise, since both derivations were based on the same image formation model and the same statistical assumptions about the signal and the noise. However, Eq. (9) was derived by straightforward optimization of the posterior distribution and does not involve any arbitrary decisions. As is typical for parameter estimation inside the expectation–maximization algorithm, both the power of the noise and the power of the signal are learned from the data in an iterative manner through Eqs. (10) and (11), respectively. The result is that Eq. (9) will yield an estimate of *V* that is both CTF corrected and low-pass filtered, and in which uneven distributions of the orientations of the experimental images are taken into account. As such, to my knowledge, this expression provides the first implementation of the intended meaning of the Wiener filter in the case of 3D reconstruction.

The relative contribution of the two additive terms in the denominator of Eq. (9) also gives an objective indication of the SNR at any point in the 3D Fourier transform of the resulting reconstruction. Under the assumptions made above, for Fourier components where both terms are equal, the power of the noise in the reconstruction is expected to be as high as the power of the signal, that is, SNR = 1. Again, the statistical approach yields a result that is similar but not equivalent to that of existing approaches. The ratio of these two terms is most similar to the previously defined 3D spectral signal-to-noise ratio^23^ but provides additional insights into how to take the CTFs into account. To avoid confusion with previously reported SSNR definitions, I will use the notation SNR_*l*_^MAP^, for SNR in the MAP estimate. Straightforward rewriting yields the following expression:

(13) $${\text{SNR}}_{l}^{\text{MAP}}=\frac{\tau_{l}^{2}}{\sum_{i=1}^{N}\int_{\phi }\Gamma_{i\phi }^{\left(n\right)}\sum_{j=1}^{J}P^{\phi_{lj}^{T}}\frac{{\text{CTF}}_{ij}^{\text{2}}}{\sigma_{ij}^{2}}\text{d}\phi }$$

The SNR_*l*_^MAP^ yields a resolution estimate that varies in 3D Fourier space (i.e., with *l*), depending on the power of the signal, the power of the noise, the CTFs, and the orientational distribution of the 2D experimental images. However, often, a single value for the resolution of a given reconstruction is preferred. Therefore, the resolution-dependent spherical average of SNR_*l*_^MAP^ may be useful. I will refer to this spherical average as the SSNR^MAP^ and propose the highest resolution at which SSNR^MAP^ > = 1 as an objective resolution criterium for a structure determined by MAP estimation.

The iterative use of Eqs. (9–11) deserves further attention. The values of τ*_l_*^2(*n*)^ are calculated directly from the squared amplitudes of *V*_*l*_^(*n*)^ and then used to calculate *V*_*l*_^(*n* + 1)^ in the next iteration. For those *l* where SNR_*l*_^MAP^ is large, *V*_*l*_^(*n* + 1)^ will be calculated as a weighted sum over the 2D experimental images, much like the unregularized ML methods or the reconstruction in Eq. (2). For those *l* where SNR_*l*_^MAP^ is small, the amplitudes of *V*_*l*_^(*n* + 1)^ will be effectively dampened. If refinement is started from a strongly low-pass filtered reference structure, τ*_l_*^2(1)^ (and thus SNR_*l*_^MAP^) will only be large for the lowest frequency terms. Dampening of all higher-resolution terms will therefore result in relatively low-resolution estimates of *V* during the initial iterations. Nevertheless, the resolution of the reconstruction may gradually improve, provided that the SNR in the experimental images is high enough and enough iterations are performed. At some point in the iterative process, the resolution will stop improving because averaging over the noisy higher-resolution Fourier components no longer yields sufficiently high values of SNR_*l*_^MAP^.

There remains one problem with the direct implementation of Eqs. (9–11). Their derivation depends on the assumption of independence between Fourier components of the signal. This assumption is known to be a poor one because the signal, a macromolecular complex, has a limited support in real space. Consequently, the power in the signal will be underestimated, and the reconstruction will be oversmoothed. Because the assumptions of independence are crucial in the derivation of a computationally tractable algorithm, heuristics seemed the only reasonable solution to this problem. Therefore, in the calculations presented below, all estimates for τ*_l_*^2^ were multiplied by a constant, *T* = 4, in an attempt to account for the correlations between Fourier components in the signal. As expected, values of *T* close to 1 were observed to yield reconstructions with suboptimal resolutions, whereas for values larger than four, noticeable amounts of overfitting were observed (results not shown). One could argue that heuristics in existing approaches have been traded for a similar heuristics in the statistical approach. However, the heuristics proposed here are clearly argued as a consequence of limitations in the adopted statistical assumptions, whereas the reasons for heuristics in existing implementations are often arbitrary. In addition, whereas the heuristics in other approaches often involve multiple parameters, the heuristics employed here involve only a single constant whose optimal value is not expected to change much for different data sets.

## Results

The MAP approach was tested in three different scenarios, each comprising a different cryo-EM data set. The first scenario represents an extreme case of reconstruction from images of suboptimal quality and illustrates the potential pitfalls of undetected overfitting. The second scenario comprises a data set of typical size and quality and illustrates the potential benefits of the statistical approach for data that could nowadays be collected in many cryo-EM laboratories. The third scenario illustrates the effectiveness of the statistical approach in dealing with structurally heterogeneous data sets, that is, when more than one different structures are present.

### Reduced overfitting of data with low SNRs

**Fig. 2:**
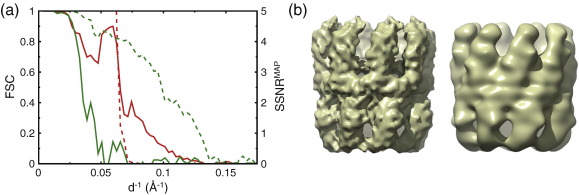
Thermosome test case. (a) Resolution estimates for the MAP (red) and XMIPP (green) refinements. Broken lines indicate resolution estimates as reported by the refinement program. The broken red line indicates the SSNR^MAP^ values for the MAP refinement; the broken green line indicates the FSC values as estimated inside XMIPP by splitting the entire data set into two random halves at the final refinement iteration. Continuous lines indicate FSC values between two independently refined reconstructions. Each of these reconstructions was refined against two completely separate random halves of the data. (b) Reconstructed maps from the XMIPP (left) and MAP (right) refinements.

The first test data set comprised 8403 archaeal thermosome particles. In a previous study, these data were judged to be of too low quality to allow reliable structure determination (Yebenes *et al*., unpublished data). Still, reference-free class averages showed 8-fold symmetric top views as well as asymmetric side views. Combination of these images led to an initial 3D map at 50 Å with C8 symmetry, and this symmetry was imposed during subsequent refinements. The initial map was first subjected to conventional refinement as implemented in the XMIPP package.^24^ This implementation merely represents one of many other available implementations for cryo-EM reconstruction and is not expected to perform significantly better or worse than most of them. It comprises standard projection matching in polar coordinates, reconstruction by direct Fourier inversion, regularization by low-pass and Wiener filtering, and resolution estimation by Fourier shell correlation (FSC) between reconstructions of random halves of the data at every iteration. Based on the FSC = 0.5 criterion, the resulting reconstruction was estimated to have a resolution of 10 Å (Fig. 2a, broken green line), which might have been considered a reasonable result given that over 65,000 asymmetric units had been averaged. However, further analysis of the map revealed indications of severe overfitting, most notably a typical “hairy” aspect of the density, that is, with many high-resolution features superimposed on a low-resolution ghost of the initial model. In addition, the map lacked features one would expect at this resolution, for example, the presence of rod-shaped densities for α-helices (Fig. 2b, left). The presence of overfitting was confirmed by two completely independent refinements of random halves of the data that were started from the same initial model. Whereas these refinements yielded reconstructions with estimated resolutions of 11 and 12 Å, respectively, the two resulting maps correlated with each other only up to 30 Å (Fig. 2a, continuous green line). At this point, it should be noted that this degree of overfitting could probably have been avoided by careful low-pass filtering of the images prior to refinement and/or tuning of the parameters of the refinement protocol itself. However, such procedures were not performed in the MAP refinement described below, and they were deliberately omitted from the XMIPP refinement in order to illustrate the potential pitfalls of nonexpert use of conventional refinement strategies.

Refinement of the same model by the MAP approach yielded a reconstruction for which the SSNR^MAP^ dropped below 1 at 16 Å (Fig. 2a, broken red line) and which did not show strong indications of overfitting (Fig. 2b, right). In this case, independent refinements of two random halves of the data resulted in reconstructions that both had an estimated resolution of 16 Å and which also correlated with each other up to 16 Å (Fig. 2a, continuous red line). Further analysis revealed that a dip at 20–30 Å resolution in the FSC curve between the two independently refined maps could be related to the observation that a majority of the CTFs passed through zero close to 30 Å resolution. Probably, due to a scarcity of experimental data at this resolution, overfitting was not completely abolished in the statistical approach. Still, compared to the conventional approach, overfitting was significantly reduced, resulting in a better map and a more reliable resolution estimate.

### Increased objectivity in map interpretation

**Fig. 3:**
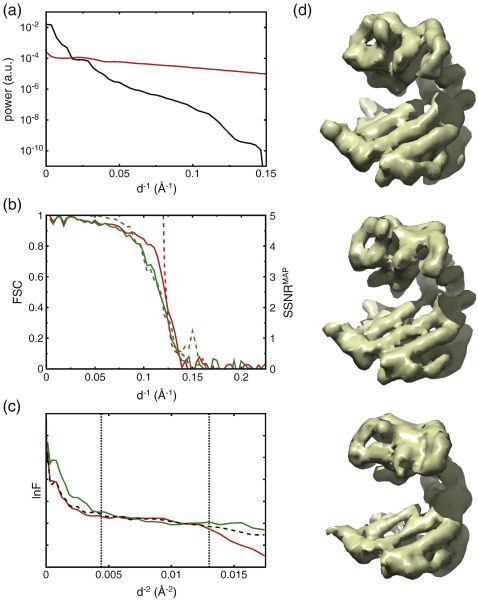
GroEL test case. (a) Spherical average of the power of the signal (τ*_l_*^2^, in black) and the annular average of the power of the noise in one of the micrographs (σ*_ij_*^2^, in red) as estimated by MAP refinement. (b) Resolution estimates for the MAP (red) and XMIPP (green) refinements. Broken lines indicate resolution estimates as reported by the refinement program. The broken red line indicates the SSNR^MAP^ values for the MAP refinement; the broken green line indicates the FSC values as estimated inside XMIPP by splitting the entire data set into two random halves at the final refinement iteration. Continuous lines indicate FSC values between the reconstructions and the crystal structure (see also Experimental Procedures). (c) Guinier plots for the atomic model (black) and the sharpened reconstructions from the MAP (red) and XMIPP (green) refinements. Vertical dotted lines indicate the resolution range that was used to estimate the *B*-factor for sharpening the experimental reconstruction, using the atomic model as a reference. (d) Density maps for the atomic model at 8 Å resolution (top) and the sharpened reconstruction from the MAP (middle) and XMIPP (bottom) refinements.

The second test data set comprised 50,000 unliganded GroEL particles that were randomly selected from an original data set of 284,742 particles.^25^ After sorting and analysis of 2D class averages, 39,922 particles were selected for 3D reconstruction using either MAP estimation or conventional refinement in XMIPP. Refinements were performed imposing D7 symmetry, and a starting model was obtained by applying a strict  50-Å low-pass filter to the 7.8-Å reconstruction that was reported for the original data set (Electron Microscopy Data Bank ID: 1200). MAP refinement yielded a reconstruction for which the SSNR^MAP^ dropped below one at a resolution of 8.0 Å (Fig. 3a, broken red line). Conventional projection matching in XMIPP gave a reconstruction with an estimated resolution of 8.8 Å (Fig. 3a, broken green line). The calculation of FSC curves between these maps and a fitted 2.9-Å GroEL crystal structure (Protein Data Bank ID: 1XCK, see Experimental Procedures) confirmed that MAP refinement had reached a higher resolution than the conventional approach (Fig. 3a, continuous lines). The favorable comparison in resolution with the XMIPP reconstruction (and with the reconstruction that was reported for the originally much larger data set) indicates that regularization with a Gaussian prior does not result in oversmoothing of the reconstruction. On the contrary, through optimal filtering of the reconstruction during the refinement, higher resolutions may be obtained than with conventional approaches.

Prior to visualization, the reconstructed density maps were sharpened using the approach proposed by Rosenthal and Henderson.^16^ Through the use of the density map generated from the crystal structure as a reference, which itself has an estimated *B*-factor of 250 Å^2^, application of this procedure led to estimated *B*-factors of 560 Å^2^ for the XMIPP-generated reconstruction and 715 Å^2^ for the reconstruction from the MAP approach. Analysis of the corresponding Guinier plots (Fig. 3b) shows that the power of the XMIPP-generated map is too strong both at low resolution and at high resolution. This suboptimal weighting of different resolutions may be attributed to heuristics employed in the Wiener filter. XMIPP uses a constant for the SNR term in the Wiener filter and sets its value in the same way as FREALIGN does.^26^ As also mentioned above, a single value is, however, inadequate to describe the intrinsic 3D behavior of the SNR in Fourier space. The statistical approach does employ a full 3D SNR model, and the Guinier plot of the reconstruction generated by MAP refinement is in excellent agreement with the model from its lowest frequency terms almost up to its estimated resolution. At the high-resolution end, despite FSC weighting,^16^ the XMIPP-generated map still has relatively strong features beyond its estimated resolution. The FSC curve with the crystal structure (Fig. 3a, continuous green line) indicates that these features are mainly due to noise. These noise features result in an underestimation of the *B*-factor in the Rosenthal and Henderson approach. On the contrary, the signal in the map generated by the statistical approach drops sharply near its estimated resolution limit, which is a direct consequence of the low-pass filtering effects of Eq. (9). Therefore, whereas interpretation of the XMIPP-generated map at too high resolutions would be subject to errors, interpretation of the reconstruction from the statistical approach is unambiguous. Comparison of the sharpened reconstructions with an  8-Å low-pass filtered map that was generated from the crystal structure confirms the good quality of the MAP reconstruction and illustrates the problems of the conventional approach (Fig. 3c).

### Classification of minority conformations

**Fig. 4:**
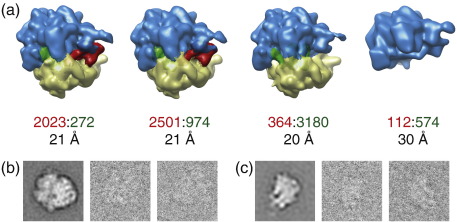
Ribosome test case. (a) Reconstructed maps for a MAP refinement with *K* = 4 classes. Density for EF-G is shown in red, 50S subunits are shown in blue, 30S subunits are shown in yellow, and tRNAs are shown in green. The first two maps were interpreted as 70S ribosomes in complex with EF-G, the third map was interpreted as a 70S ribosome without EF-G, and the fourth map was interpreted as a 50S ribosomal subunit. For each class, the numbers of assigned particles that according to supervised classification correspond to ribosomes with EF-G and without EF-G are indicated in red and green, respectively. (The true class assignments are not known.) In addition, the resolution at which SSNR^MAP^ drops below 1 is indicated for each class. (b) A representative reference-free class average (left) and two individual, unaligned experimental images (right) for particles assigned to the class corresponding to 70S particles without EF-G. (c) As (b), but for the class corresponding to 50S subunits.

The third test data set comprised 10,000 *Escherichia coli* ribosome particles that were proposed as a benchmark for 3D classification algorithms.^27^ Supervised classification had previously suggested that 5000 of these particles correspond to ratcheted ribosomes in complex with elongation factor G (EF-G) and a single tRNA molecule, while the other 5000 particles were interpreted as unratcheted ribosomes without EF-G and in complex with three tRNAs. Various classification algorithms have been tested using this data set, and all of them have reported results similar to the ones obtained using supervised classification.^28–31^ However, simultaneous refinement of *K* = 4 reconstructions in the MAP approach (see also Experimental Procedures) identified a third previously unobserved class. Whereas, as expected, the first two maps of this refinement were interpreted as 70S ribosomes in complex with EF-G, and the third map as a 70S ribosome without EF-G, the fourth map corresponded to a 50S ribosomal subunit (Fig. 4a). A second calculation with randomly different initial models yielded similar results, with a 94% overlap in the 50S class. Although this class contains only a small minority of the particles (i.e., 6%), visual inspection of these particles and their reference-free 2D class averages confirmed the existence of 50S particles in the data (cf. Fig. 4b and c). Note that, as expected, the effective resolution as measured by the SSNR^MAP^ is much lower for the minority class (30 Å) than for the other three classes (20–21 Å), which is a direct consequence of the lower number of particles contributing to the term on the left-hand side of the denominator of Eq. (9). The absence of such class-specific regularization is likely to lead to very noisy reconstructions for small classes in existing classification approaches, which may explain their failure in identifying the 50S class.

As in related ML classification approaches,^6^ the number of classes *K* is assumed to be known, that is, this number needs to be provided by the user, but this assumption is hardly ever met. Often, comparing calculations with different values of *K* provides a useful band-aid, but admittedly, there is no well-established, objective criterion to decide on its optimal value. In this case, refinements with *K* = 3 were not successful in revealing the 50S class, but refinements with *K* = 5 did give results similar to the ones in Fig. 4, albeit with an additional class corresponding to the 70S ribosome without EF-G (results not shown).

## Discussion and Conclusions

Because the accumulation of noise in cryo-EM reconstructions is a consequence of the ill-posed character of the reconstruction problem, which in turn is caused by the high noise levels and the incompleteness of the experimental data, one could discern three general ways of improving cryo-EM reconstructions. Firstly, lower noise levels in the data will reduce ill-posedness and thus lead to better reconstructions. In this light, ongoing developments to improve microscopes (e.g., see Ref. 32) and detectors (e.g., see Ref. 33) are expected to make an important contribution to the field. Secondly, reducing incompleteness (due to unknown relative orientations) will also reduce ill-posedness and thus lead to better reconstructions. Obvious examples of less incomplete reconstruction problems are those where the molecules adopt some kind of internal symmetry, for example, helical assemblies or 2D crystals. It is therefore not surprising that, in particular for those systems, cryo-EM has been most successful in terms of resolution and map quality,^34^ but also for reconstruction of asymmetric single particles, one might devise modifications of existing sample preparation protocols that somehow provide information on the orientations of the particles and thus reduce incompleteness (e.g., see Ref. 35) Thirdly, and this is the approach that has been taken in this paper, ill-posedness may be reduced by regularization, that is, the incorporation of prior information in the refinement.

In this paper, the use of smoothness has been explored as a source of prior information about cryo-EM reconstructions. However, the observation that overfitting was not completely abolished in the thermosome example illustrates that smoothness alone might not be sufficient to fully determine a unique structure from very noisy cryo-EM data. One could envision the use of additional, more powerful sources of prior knowledge, such as non-negativity, solvent flatness, or ultimately the large amount of chemical knowledge that is available about proteins and nucleic acids. It might also be possible to identify alternative sources of prior knowledge from existing approaches that are aimed at reducing overfitting, such as that of Stewart *et al*.^36^ The statistical framework described in this paper may be used to combine any source of prior information with the experimental data, provided that suitable numerical expressions may be formulated. In addition, it is foreseeable that the heuristics employed in this paper to prevent oversmoothing (multiplication of the estimates for τ*_l_*^2^ with a constant) may be improved in the future. More detailed analyses of the correlations between Fourier components of macromolecules or the use of power spectra of previously determined structures may lead to better estimates for τ*_l_*^2^. Meanwhile, reconstructions obtained by MAP refinement should report the value of *T* employed, and values much larger than 4 should probably be avoided.

In general, the Bayesian view provides a rigorous theoretical framework for cryo-EM single-particle reconstruction, in which the explicit statistical assumptions can be criticized and, if possible, modified to provide better reconstructions. The procedures presented here render commonly employed heuristics in low-pass and Wiener filtering largely superfluous, as Bayes' law uniquely determines how observed experimental data should be combined with prior knowledge. As such, the Bayesian approach leaves little scope for arbitrary decisions by the user, which will alleviate the need for user expertise and ultimately contribute to increased objectivity in the reconstruction process.

## Experimental Procedures

### Cryo-electron microscopy

Thermosome complexes from the hyperthermophylic archaeum *Thermococcus* strain KS-1 containing only α-subunits^37^ were imaged under low-dose conditions in a FEI T20 microscope at 200 kV and a magnification of 50,000×. Micrographs were recorded on photographic film, scanned using a Zeiss SCAI scanner with a pixel size of 7 μm, and subsequently downsampled by a factor 2. Particles were picked manually and extracted in boxes of 120 × 120 pixels with a resulting pixel size of 2.8 Å.

The GroEL data set used here is a random subset of the 284,742 particles described by Stagg *et al*.^25^ In that study, data were collected in an automated manner using Leginon^38^ on a FEI T20 microscope that was operated at 120 kV, and images were recorded on a 4*k* × 4*k* Gatan Ultrascan CCD at a magnification of 50,000×. Particles were selected automatically using template-based procedures and extracted in boxes of 128 × 128 pixels with a pixel size of 2.26 Å.

The ribosome data set used here is a subset of the 91,114 particles described previously^21^ and was downloaded from the Electron Microscopy Data Bank†. In this case, *E. coli* ribosomes in a pre-translational state were imaged under low-dose conditions on a FEI T20 electron microscope at 200 kV with a calibrated magnification of 49,650×. Particles were selected by preliminary automated particle picking, visual verification, and subsequent selection based on cross-correlation coefficient with a template. Particles were extracted in boxes of 130  × 130 pixels with a pixel size of 2.8 Å. Supervised classification had previously suggested that 5000 of the 10,000 particles used here correspond to unratcheted ribosomes without EF-G, and the other 5000 particles, to ratcheted ribosomes in complex with EF-G.

### Implementation

The iterative algorithm in Eqs. (9–11) was implemented in a stand-alone computer program called RELION (*RE*gularised *LI*kelihood *O*ptimisatio*N*), which may be downloaded for free online‡. Although, for the sake of clarity, Eqs. (9–11) do not describe the case of simultaneous refinement of *K* different 3D models, derivation of the corresponding algorithm is straightforward. Moreover, the same theory may be used to derive the algorithm that simultaneously refines *K* 2D models. RELION implements both the 2D and the 3D cases of multi-reference refinement, and as such may be used for 3D classification of structurally heterogeneous data sets, as well as the calculation of 2D class averages.

As was recognized by Sindelar and Grigorieff,^39^ the power of the noise estimated from unmasked images is higher than that estimated from images that are masked to the area where the actual particle resides. Therefore, if unmasked images were used, this would lead to an overestimation of the noise by Eq. (10) and thus oversmoothing of the maps by Eq. (9). On the other hand, the use of masked images would lead to correlations between the assumedly independent Fourier components. All calculations presented in this paper were done using unmasked images, but an option to use masked images has also been implemented.

Whereas Eq. (10) implies that the power of the noise is estimated as a 2D array for each experimental image, in practice, estimates for the power of the noise are obtained by averaging σ*_ij_*^2^ over resolution rings and groups of images, for example, all images from a single micrograph. Also, estimates for the power of the signal, that is, τ*_l_*^2^, are obtained by averaging over resolution shells. Note that, despite this averaging, SNR_*l*_^MAP^ still varies in 3D depending on the orientational distribution of the images. Also, for the sake of simplicity, Eq. (9) does not reflect the corrections that are needed to account for interpolation operations and nonuniform sampling of the 3D Fourier transform. In practice, the 3D transform is oversampled three times, and projections as well as back-projections are performed by nearest-neighbor interpolation. An iterative gridding approach^40^ is then used to deal with the nonuniform sampling of the oversampled 3D transform, prior to calculation of the inverse Fourier transform.

### Image processing

All other image processing operations were performed in the XMIPP package.^18^ Prior to refinement, all data sets were normalized using previously described procedures.^5^ MAP refinements and projection matching refinements in XMIPP were performed with similar settings where possible. Although the implementation of the MAP approach readily handles anisotropic CTF models, all refinements were performed with isotropic CTFs (without envelope functions) for the sake of comparison with XMIPP. All orientational searches, or integrations in the statistical approach, were performed over the full five dimensions, that is, three Euler angles and two translations. For both the thermosome and the GroEL refinements, the first 10 iterations were performed with an angular sampling of 7.5°, and subsequent iterations were performed with an angular sampling interval of 3.75°. Thermosome refinements were stopped after 15 iterations, and GroEL refinements, after 20. Translational searches were limited to ± 10 pixels in both directions in the first 10 iterations and to ± 6 pixels in the subsequent iterations. Although it is common practice in XMIPP to reduce computational costs by breaking up the orientational search into separate rotational and translational searches and to limit rotational searches to local searches around previously determined orientations, this was not done in the refinements presented here for the sake of comparison with the MAP approach. Refinements with angular sampling intervals as fine as 1° where such tricks were employed did not result in better reconstructions (results not shown).

The true resolution of the GroEL reconstructions was assessed by FSC with a published crystal structure (Protein Data Bank ID: 1XCK). This structure contains 14 unique monomers in its asymmetric unit. Each of these monomers was fitted separately into the reconstructions using UCSF Chimera,^41^ and for each monomer, the equatorial, intermediate, and apical domains were allowed to move independently as rigid bodies. The resulting coordinates were converted to an electron density map that was symmetrized according to *D*7 symmetry. Optimization of the relative magnification between this map and the cryo-EM reconstructions revealed that the effective pixel size of the cryo-EM images was 2.19 Å, differing by 3% from the nominal value, and this value was used to generate all plots in Fig. 3.

Ribosome refinements were performed for 25 iterations with an angular sampling of 7.5° and translational searches of ± 10 pixels. To generate *K* = 4 unsupervised initial starting models from a single  80-Å low-pass filtered initial ribosome structure, during the first iteration, we divided the data set into four random subsets in a way similar to that described before.^21^

## Footnotes

† http://www.emdatabank.org/emtestdata.html

‡ http://www2.mrc-lmb.cam.ac.uk/relion
